# Empowering Public Health Nurses and Community Home Visitors through Effective Communication Relationships

**DOI:** 10.3390/nursrep11030062

**Published:** 2021-08-28

**Authors:** Debbie Sheppard-LeMoine, Megan Aston, Lisa Goldberg, Judy MacDonald, Deb Tamlyn

**Affiliations:** 1 Faculty of Nursing, University of Windsor, Windsor, ON N9B 3P4, Canada; debbiesl@uwindsor.ca; 2 School of Nursing, Dalhousie University, Halifax, NS B3H 4R2, Canada; Lisa.Goldberg@Dal.Ca (L.G.); Judy.MacDonald@Dal.Ca (J.M.); dltamlyn@dal.ca (D.T.)

**Keywords:** family, family nursing, community, public health nursing, health visiting, women, feminist, discourse analysis, qualitative

## Abstract

Home visiting programs for marginalized families have included both Public Health Nurses (PHNs) and Community Home Visitors (CHV). Support for families requires health care providers to implement effective communication and collaboration practices; however, few studies have examined how this is carried out. The purpose of this qualitative research study was to explore how an Enhanced Home Visiting (EHV) program in Nova Scotia Canada was organized, delivered through the experiences of PHNs and CHVs. Feminist post-structuralism informed by discourse analysis was used to understand how their experiences were socially and institutionally constructed. Individual semi-structured interviews were conducted with 6 PHNs and 8 CHVs and one focus group was held with 10 of the participants. A social discourse on mothering layered within a social discourse of working with a vulnerable population added a deeper understanding of how communication was constructed through the everyday practices of PHNs and CHVs. Findings may be used to inform reporting and communication practices between health care providers who work with marginalized families.

## 1. Introduction

This article presents findings from a research study that examined the experiences of public health nurses (PHNs) and community home visitors (CHVs) working within a Canadian public health Enhanced Home Visiting (EHV) program for mothers identified as vulnerable. In particular, we will focus on how different communication styles affected the practices of PHNs and CHVs as they negotiated relations of power within their practice.

Home visiting programs designed for families identified as disadvantaged due to socioeconomic and other inequities have been available since the 1800s [1]. These programs can be found internationally [2] and many research studies [3,4,5] and systematic reviews [6] can be found reporting on the effectiveness of such programs. The Nurse-Family-Partnership is one globally renowned program that has been taken up across many countries [7]. While many programs are nurse-led, some programs may also include health care providers who are not nurses.

In Nova Scotia Canada, PHNs have a long history of delivering universal home visiting programs for all parents such as the healthy beginnings program and more recently, the targeted EHV program. Community home visitors (CHV) are often an integral part of targeted family support in the early years soon after the birth of a newborn up to the age of preschool. This refers to families that may be at risk for poor health outcomes. The language of universal and targeted is commonly used in association with home visiting [8,9]. In Nova Scotia, universal refers to home visiting offered to all families, and enhanced or targeted refers to home visiting offered to a specific population of families who have distinct needs such as those screened into the EHV program in Nova Scotia (Healthy Beginnings Home Support Handbook). ‘Targeted’ usually refers to programs designed to support families experiencing socioeconomic distress, mental health issues, lone parenting, and other hardships [9,10,11,12,13,14]

In response to the identified need to support vulnerable families such as mothers parenting on their own and experiencing burdens from low incomes, the Canadian Government established the federally funded Healthy Beginnings program with a focus on supporting the health of families with children up to 3 years of age. In Nova Scotia, the Enhanced Home Visiting (EHV) program began in 2002 for families with newborns requiring additional support. These families are assessed as at risk of poor health outcomes due to challenging life circumstances [15] and are offered services from public health nurses and community home visitors. The work of PHNs and CHVs is integral to the success of these programs and warrants an in-depth examination of how they deliver services.

### Background

The health of mothers and that of their families is influenced primarily by the economic and social conditions they experience in their daily lives [16,17]. Public Health Nurses (PHNs) have a historic tradition of home visiting that supports the health of the most marginalized families including mothers who have been affected by their economic and social conditions. In a Canadian study that included 32 PHNs, Browne et al. argued that while there is documented evidence that PHNs support the health of vulnerable families, it is difficult to find studies that focus on how PHNs do their supportive work.

Home visiting programs vary across Canada. However, currently, in Nova Scotia one home visiting program includes both PHNs and Community Home Visitors (CHVs) who work within the Nova Scotia EHV program for families requiring extra support with their newborn at home until they reach 3 years of age. Changes to universal home visiting programs occurred in Nova Scotia in 2002 when CHVs (para professionals) were added to create a more targeted program that supported mothers and their families needing extra support parenting and promoting optimal development and health of their children at home as part of the Healthy Beginnings EHV [15]. Many of the mothers in the program are single mothers however other families in the program include those who have experienced difficult life circumstances that put extra burdens on a family caring for a newborn baby. Examples of difficult circumstances include: geographic isolation from services; limited education; lack of support from families, friends, or community; personal experiences with mental health issues; and other unique burdens specific to a family where a need is identified by an in-depth assessment [15]. While the EHV program is inclusive of mothers and families who experience extra burdens that may impact their parenting of a newborn it is important to understand the impact that economic conditions have on family experiences caring for a newborn. As one of the major determinants of health, low income, and in many cases poverty has a significant impact on families because of existing social circumstances families with extra burdens in the enhanced home visiting program are already experiencing. Mothers, especially those who are parenting on their own and who experience low incomes, have the poorest health outcomes in Nova Scotia and across Canada and are directly affected by practices of CHVs and PHNs within the EHV as participants in this public health program [17].

The work of PHNs requires them to understand the personal and social issues in health within the context of their practices with mothers and their families [18,19]. In home visiting, the nurse–client relationship has been considered a major factor that supports the success of home visiting with mothers and families requiring extra support for diverse reasons [20]. Currently, relationships are developed among PHNs, CHVs, mothers, and their families as part of the Nova Scotia Enhanced Home Visiting Program [15]. While there have been informative evaluations carried out on the Nova Scotia enhanced home visiting program, a deep understanding of the personal and social practices including communication of both CHVs and PHNs that surround mothers and families is not evident in the reports. In Nova Scotia, community home visitors (CHVs) were added as the primary home supporters of families who were accepted into the EHV program. The CHVs came from diverse backgrounds and levels of education and were usually non-professionals. The addition of CHVs was an enhanced change to home visiting practices that had previously been conducted exclusively by PHNs when supporting Nova Scotia families with children up to 3 years of age (K. Inkpen, Personal Communication, Nov. 2012). No research was found that focused on the experiences or communication between PHNs and CHVs who worked within home visiting programs.

Upon reflection of this gap in a Nova Scotia context, we believed it was important to implement a study that would examine the personal, social, and institutional practices of PHNs and CHVs who worked within a home visiting program for vulnerable mothers and their families using feminist poststructuralism informed by discourse analysis.

#### Research Purpose and Questions

The purpose of this research was to explore how the Nova Scotia Enhanced Home Visiting program for vulnerable mothers and their families was organized, delivered, and experienced through the practices and experiences of PHNs and CHVs in Nova Scotia. The analysis provided critical information about how communication practices and experiences of PHNs and CHVs affected how services were delivered as well as how communication was deeply rooted within personal, social, and institutional discourses that represented the enhanced home visiting program. There is potential for this study to provide decision and policymakers with another way to understand the known, unspoken, and hidden communication practices that support vulnerable mothers and their family’s health in the Nova Scotia EHV Program.

The research questions were: 1. How are Enhanced Home Visiting services understood and experienced by PHNs? 2. How are Enhanced Home Visiting services understood and experienced by CHVs? 3. How are Enhanced Home Visiting services communicated between PHNs and CHVs?

Guided by a feminist poststructuralist (FPS) methodology [21,22,23,24,25,26,27,28] this inquiry revealed how power operated through discourse to create knowledge about the social and institutional construction of home visiting with vulnerable mothers and families who experience difficult life circumstances such as: lack of access to services, limited education, lack of family, friend or community support, low incomes, mental health issues, or other unique experiences that socially construct their health outcomes. Uncovering both positive and oppressive conceptualizations of power through the discourses of the participants created an opportunity to redesign alternate subjective positions. Therefore, through a feminist poststructuralist lens, our understanding of EHV practices of PHNs and CHVs can guide changes in policies, knowledge, programming, and practices of both professionals and laypersons. This philosophical approach in this research inquiry promoted a complex examination of how the social, historical, and political factors influence the communication practices that support the health of mothers and families who are marginalized.

## 2. Methodology

### 2.1. Design

The theoretical orientation of the study was guided by Feminist Post Structuralist Methodology (FPS) [21,22,23,24,25,26,27,28]. Foucault is recognized for his concern with how accepted societal knowledge creation is related to power and he suggests that people’s understanding of their personal view of self is produced by the institutions that surround them. Foucault would support the view that a person is socially constructed by those institutions that surround their day-to-day life which creates a lack of individual consciousness [29,30,31]. Foucault emphasized understanding the role of discourse, power, and subjectivity throughout his work.

Intentionally, Foucault wanted to shake up what was accepted to be true in society, and while he did not focus on women’s experiences he successfully engaged some feminists as they could see ways to apply his philosophy with their approach to studying women’s experiences [23,28]. Through challenging societal assumptions about reality and truth Foucault pushes us to also consider the causes and effects of life experiences and the concept of agency in people’s lives to make decisions, change, and create a new way of experiencing the world [22,32,33,34]. Foucault deconstructed the power structures of society through the examination of institutional practices. Foucault wanted to uncover how knowledge and its related power is created in society and becomes accepted as the norm. Through uncovering how societal structures contribute to the creation of power relations, Foucault believed action through change could occur.

The philosophical underpinnings of feminism and post-structuralism create the theoretical framework of Feminist Post-structuralism [23,28] and provide a way to challenge assumptions about what is understood to be every day and potentially hegemonic practices within health care practices [21,23,24,28,35,36]. Feminist post-structuralism supports understanding and examining the structures that regulate and affect practices and services [28]. Feminist post-structuralism suggests that established meanings that are part of common societal regulation of others do not need to be taken for granted as the only normative assumption about health care practices [28]. As a framework, feminist post-structuralism can be applied to diverse social practices and facilitate uncovering meanings, values, and relations of power that control practices which are defined as those activities that people engage in during their day-to-day lives. Feminist post-structuralism as a theoretical framework addresses how the meaning, values, and relations of power are negotiated, who benefits from them, how they have maintained their power, and asks the question “is there a way to shape a new understanding of the structure in control?” [23,28].

### 2.2. Recruitment

Approval of the research study and ethics were obtained from the Nova Scotia Health Authority and Dalhousie University, Canada before recruitment began. Purposeful sampling was used in this inquiry through the recruitment of participants who were willing to share their experiences as PHNs and CHVs [37]. Participants were recruited based upon the following inclusion criteria: PHNs and CHVs who supported mothers as part of the Nova Scotia Enhanced Home Visiting Program for at least 6 months to ensure the participants had practice experiences to reflect upon. The consent forms were explained by the researcher and signed prior to participation in the interview and focus group. While anonymity could not be guaranteed, the participants were assured that confidentiality would be maintained by removing all identifying information. Pseudonyms were assigned to each participant. Audiotapes and files were stored in a locked filing cabinet and all electronic files were password protected. It was reinforced by the participants that they could withdraw from the study up until one month after they had been interviewed and they could refuse to answer any questions at any time. In addition, if the participants had any questions that needed answering throughout the research process they were encouraged to ask. Recruitment, data collection, and data analysis occurred from 2014–2015.

### 2.3. Data Collection

All participants gave written and verbal consent before beginning the data collection. The consent forms were explained by the researcher and signed prior to participation in the interview and focus group. While anonymity could not be guaranteed, the participants were assured that confidentiality would be maintained by removing all identifying information.

All of the interviews except two took place in a private, confidential space at a family resource center for the CHVs and at the public health offices for the PHNs. Two interviews were conducted through a video conferencing platform. Face-to-face, semi-structured interviews were conducted with each participant. Interviewing from a feminist perspective involves listening to the participants’ experiences from their perspective and in their own words so as to understand personal meanings. The interviews were facilitated to be non-hierarchical and more conversational between the researcher and the interviewee [38]. Reflexive awareness was maintained by the researcher which included acknowledging that the interview is a complex encounter where deep-rooted feelings of identity, power, culture, and constructions of feelings similar or different shape the interview experience [38]. Interviews were 60–90 min at a location and time agreed upon by the participants and researcher. All interviews were audio-recorded and then transcribed verbatim. Pseudonyms were assigned to each participant. It was reinforced to the participants that they could withdraw from the study up until one month after they had been interviewed and they could refuse to answer any questions at any time. In addition, if the participants had any questions they needed answering throughout the research process they were encouraged to ask.

The preliminary study findings were presented to the participants for their input in a focus group. A summary of the main themes was shared with 6 CHVs and 4 PHNs who decided they would like a combined focus group. The participants agreed that the preliminary study findings represented the messages they wanted to publicly share. The focus group discussion was audio-recorded and transcribed verbatim. Audiotapes and files were stored in a locked filing cabinet and all electronic files were password protected.

### 2.4. Data Analysis

Discourse analysis was used to analyze the transcripts. Beliefs, values, and practices were identified which then guided the analysis to understand how personal negotiations of power were constructed through social and institutional discourses. Aston [21] provides a discussion and guide of this process. Subjectivity and agency were also incorporated into the analysis. A comparison of themes within and between interviews also occurred.

## 3. Results

Discursive representations of 6 PHNs and 8 CHVs’ practices were made visible and a deeper understanding of their communication experiences and practices was revealed that were related to vulnerable mothers within EHV in Nova Scotia. Four sub-themes emerged: (1) Reflective practice needs supportive communication, (2) shifting the taken-for-granted communication hierarchy, (3) building relationships through strengths-based communication, and (4) negotiating transitions of mothers/families from PHNs to CHVs.

### 3.1. Reflective Practice Needs Supportive Communication

All of the PHNs and CHVs spoke about the importance of reflecting upon their EHV practices and speaking directly with peers and or supervisors. It was important for them to speak directly because most of their practice was spent working independently in the community with mothers.

It is important to note that differences emerged between the way PHNs and CHVs experienced reflective practice. The majority of PHNs spoke about how they would debrief with another colleague about their home visiting experiences but rarely spoke with a supervisor. CHVs talked about how they were required to meet with their supervisor at the Family Resource Center weekly where they reflected with their supervisor what they had experienced in their home visiting practices. It was a time for the supervisor to hear about the experiences and to supervise the CHV.

Tory, a CHV spoke about how she valued speaking with her supervisor as she felt it supported her in her practice. She said “…breaking it down with your supervisor and going through it…being able to process it…you have to talk about things to process it…sometimes you just need to say it and put it in its place.”

Alexandria another CHV shared how reflection was part of the type of communication she appreciated in her practice. “And even to have that space with a supervisor to just have kind of a non-judgemental debrief…sometimes you come out of a visit and you’re like you’re so overwhelmed…so she understands what home visiting is like.”

Sara, another CHV also valued being supported through reflective communication with her supervisor as well as the PHN who was the coordinator of the EHV program.

“Having one consistent coordinator that we could all go to, so someone that, you know, if I go to my supervisor who’s there, you know, and she doesn’t know how to support me, she can go to that one coordinator and get the support that she needs in order to be able to in turn give me the support and vice versa. That I can go to that person. So I guess that’s what I valued the most.”

As part of a hierarchical relationship, Sara was expected to meet with her supervisor weekly to share what she had done in her home visiting practices. Sara was subjectively positioned in relation to her supervisor to ‘report’ to her, debrief and tell her how things were going. From Sara’s perspective, she described this hierarchical relation as a positive experience. We can see that this particular negotiation of power was beneficial for both parties and it appears that Sara felt empowered and supported in her role as a CHV.

We can see that the communication interactions between Sara and her supervisor differed when compared to communication between Sara and the PHN coordinator. The PHN in this role worked for Public Health but she was not part of the Family Resource Center where the supervisor worked. Sara valued the coordinator position because she believed that when everyone (PHNs and CHVs) communicated with one EHV coordinator it resulted in everyone being able to support each other through sharing with one consistent supportive coordinator. Sara felt supported by the coordinator as she believed it was a non-hierarchical relation of power and believed that other CHVs and PHNs also experienced this positive relationship.

During the course of the study, the coordinator position was eliminated. All participants in the study commented that this was a significant loss as they no longer had someone for whom they could talk to and debrief in a safe, non-hierarchical manner. Most of the participants shared examples of reflection as a type of communication they valued with peers, managers, or supervisors. The PHNs and CHVs described similar and different roles of reflection in their practices.

Because PHNs have a different scope of practice when working with mothers during home visits, compared to CHVs they are able to make independent decisions about their practice and are not required to debrief with a supervisor. Therefore, they described reflection in a different way. PHN Jasmine commented on reflection for herself and for CHVs.

“…the need for self-reflection and being able to go back and have someone that you are able to have that conversation with…I know who my people are that I can go to and I would have a conversation with… It is supposed to be scheduled…they’re [CHVs] supposed to have regular supervision once a week and that opportunity to have that relationship…”

Although PHNs were not required to report to or debrief with a supervisor or the PHN coordinator, Aggie, a PHN, expressed her belief about the importance of reflecting on her practice with others. The following quotation represents the beliefs of all the PHNs in the study.

“My managers, coordinator and practice lead, they’re all really important to me. To feel supported, to feel they believe in me, sometimes affirmation helps as well. Like I show something and, you know, we can kind of celebrate some of those success stories. Having someone you can share things with because they’re part of your team…I see the whole team approach as being really important…”

It is important to note the way Aggie described her relationship with managers and the PHN project lead. She used the words ‘affirmation’ ‘team’ ‘celebrate successes’ ‘someone you can share with’. Although there was a difference between PHNs and CHVs in the way they debriefed with their supervisors, managers, and PHN coordinator they all felt very positive about their interactions and valued the opportunity to reflect upon their practice.

The relation of power between the PHN and the person they reflected with seemed equal and represented more of a sharing of information. CHVs were expected to discuss their practice with their supervisor weekly. In the practices of CHVs, reflection was regulated by the dominant health institutional discourse represented by the supervisor at the Family Resource Centre. An obvious hierarchical relation of power between the CHV and their supervisor was established as an expectation from the institution regarding how the practices of the CHVs should be monitored. Foucault (1980) would suggest that the dominant societal structure that created a reflection in the communication practices of CHVs also regulated how it was experienced by CHVs during their weekly reflections. While the CHVs described reflection as part of their communication practices differently from PHNs, they believed that they benefitted from the reflection in similar ways to PHNS.

The importance of reflection in EHV practice was an expressed need for CHVs and PHNs. They believed they needed someone to talk to about the positive and negative experiences in a confidential way with someone who understood their practices. CHVs and PHNs worked with vulnerable families daily and they experienced some difficult situations by themselves in isolation from other colleagues.

Losing this in their practice put more burden on them to re-negotiate a way to continue reflecting with someone and in some cases with no one.

### 3.2. Shifting the Taken-for-Granted Communication Hierarchy

While the obvious difference in hierarchies between PHNs and CHVs appeared not to negatively impact their desires and abilities to debrief with supervisors, managers, and the PHN coordinator, the hierarchies did create some tensions. Sara, a CHV, expressed how she at times felt her knowledge was devalued.

“I don’t know, like I mean I think that it wouldn’t even have to be like a one-on-one situation. You know, even in a group setting as all of home visitors. Like you know, this is what we’re thinking. What do you think of that, kind of thing? Or this is a tool that we’re going to use to try to assess families. Can you look at it and see if it’s something that… I mean we’re the people that work with these moms, day in and day out. If I don’t think she’s going to answer the question, she’s not going to answer the question. And I’m not being cocky or anything like that but it’s the way that it is. So I mean we do have a lot of really valuable information that they could use, to learn which way or which direction they should go.”

Sara expressed how she felt she wasn’t being heard and alluded to a hierarchy when she said “we do have a lot of really valuable information that they could use…” Sara’s tone seemed a bit defensive when she said “I’m not being cocky…” yet she was clearly concerned that she and other CHVs, the people who were “doing the work”, were not being heard. She clearly had identified a moment of tension within the relationship between PHNs, supervisors and CHVs.

Sara did not believe that she was invited to participate in important conversations that affected her practices, and this created a hierarchical relation of power between herself and decision-makers. How she chose to negotiate this relation of power with others was through sharing information with people like PHNs and her supervisor who she saw as experts. Sara challenged the imbalance of power, or the dismissal of her knowledge by indicating that PHNs and supervisors might have a lack of knowledge that she and other CHVs could add to because they (CHVs) were the ones who were primarily working with the mothers. She was challenging the everyday practices that were socially accepted that seemingly positioned her supervisor to have more power and authority whereby the voices of CHVs were not always included.

In Sara’s next example she suggested ways to facilitate improved communication among all regardless of their position in the hierarchical structure that she describes as “top to bottom”.

“And then to have really strong communication between everyone, like from top to bottom within the whole program. So manager, PHNs, supervisors here, executive director, kind of us as home visitors. So that we actually felt like we were a team…So I think that’s kind of the most important thing, so team building things. So training where the nurses were in the same training as the home visitors, and the managers were usually the ones that were putting the training on… where we all learn together as a group and kind of to help to build that one-on-one relationship with the different people within the organization. You know, that’s all kind of gone away. That’s not important anymore or a priority maybe. I don’t know what the right word is. But I think that we’re really missing that.”

Sara was talking about the non-hierarchical relation of power she had experienced among all involved with the EHV program prior to program changes where the PHN coordinator position was cut. Sara valued a relation of power where there was a shared power relation and she believed this approach to a relation of power promoted team building and shared learning. Moreover, through a nonhierarchical relation with others, she believed that her subject position was not seen as less than anyone else in the EHV program. Instead, Sara believed this way of relating built positive relationships within the organization and that was seen as a positive experience for Sara.

Another CHV, Tory, expressed how she valued the PHN coordinator role as well and believed she communicated with PHNs in the following example:

“…so the PHN is one part…we have to link together to make the whole puzzle for families right, otherwise it doesn’t work…. So again it is the communication with PHNs. The coordinator was a great support…”

CHV Coral described how she understood her role in communicating with PHNs:

“…our role is different than a public health nurse…a public health nurse used to come to our center on a regular basis…..just seeing them more often….”

All of the PHNs talked about the importance of building a positive relationship with CHVs that would then support effective communication. Jasmine said, “I value my relationship with CHVs”. Amber said, “I like as much as possible to promote partnership between us.” Pearl said, “…level of power too that comes from the fact that our relationship with the Resource Centre is on a contractual basis so there’s that kind of position of power too…in that we hold the control to say here is your funding for the next year…” Pearl’s example highlights her belief that a binary relation of power existed between Public Health and Family Resource Centers because of funding. This in turn affected the institutional and personal relationships between PHNs and CHVs. A hierarchical relationship was established through the contract structure where public health contracted EHV out to family resource centers. This arrangement created a relation of power that affected expectations about how communication patterns were created between PHNs and CHVs. When the PHN coordinator position was removed, it negatively changed communication patterns that were seen to be supportive and inclusive.

Sara talked about a tension that existed in her organization around ‘learning together’ not being a ‘priority anymore’ but it had been in the past and she valued that type of coming together that was a way of communicating.

When examining the words used by PHNs and CHVs in their communication experiences such as ‘top to bottom’, ‘team’, ‘learn together’, ‘missing’, ‘relationship’, ‘level of power’, ‘position of power’, ‘who controls’, and ‘contract structure’ they were describing doing things in a relation of power through communication in their practice. They challenged the binary relation of power or the top-down approach of decision-making because it made them feel left out of a team that had in the past worked well together and had strong communication that supported them in their practices. With the coordinating structure gone from the EHV practices, CHV Sara’s descriptions of her beliefs about decision-making represented a loss she felt as a member of a team. Sara experienced a binary relation of power where decisions were made for her, not with her. Foucault’s philosophical way of thinking provides a way to uncover how people work through power in their work. Both PHNs and CHVs believed in and valued reflection. However, this was lost from their practices when the coordinator position was eliminated. Foucault would probably suggest that the communication practices of PHNs and CHVs were created by the practices constructed within the health care institution represented by Public Health that played a dominant role in constructing their view of self in relation to their communication practices.

The hierarchy is the binary relation that the participants identified around decision making and how decisions were communicated. The public health care institution made a decision to make program changes and it did not involve PHNs and CHVs in this process. This created a binary relation of power where decision-makers told PHNs and CHVs about the decision. However, as we can see, all of the PHNs and CHVs commented on this tension and offered the solution to go back to a more inclusive and transparent communication style between CHVs, PHNs, PHN coordinators, supervisors, and managers that was less hierarchical.

PHNs and CHVs used their agency to challenge assumptions that created the accepted multiple realities of communication in their practices. PHNs and CHVs believed and valued different things about communication in their practices compared to the institutional practices that were constructed based upon what the institution valued and believed needed to be part of the communication structure. Based upon the institutional decision to eliminate the coordinator position, the difference in what was valued about the position by the institution and the PHNs/ CHVs was magnified. Questioning and challenging the elimination of the PHN coordinator position demonstrated their agency to push for another way of experiencing or creating communication in their practices, that was less hierarchical and promoted a team approach to building relationships as part of communication practices in EHV.

### 3.3. Building Relationships through Strengths Based Communication

All of the PHNs and CHVs discussed the importance of building positive working relationships between themselves and their managers and supervisors. They also provided many examples of how their practices needed to be strength-based and non-hierarchical. The majority also valued the day-to-day formal meetings and informal (going for a coffee or lunch) conversations as a way of communicating between CHVs and PHNs, CHVs and CHVs, and PHNs and PHNs.

Some of the participants expressed a desire to help their managers understand what they were doing in home visits. They believed this was important because even though managers may have had previous frontline experience, at the time of the study they were not visiting. One CHV described managers as ‘the higher-ups’ signaling a hierarchical relation of power. A tension was noted because many CHVs and PHNs believed managers did not understand what they were doing during home visits with the mothers and families. Hope, described her beliefs about this tension in her practice in the following way:

“But I don’t think they kind of always get or remember because some of them may have been frontline workers at one point in time… So I think kind of more communication between frontline and higher ups would be beneficial. I’m not saying I want to tell them how to do their job but…maybe hearing a story about, you know a challenging visit, or you know, hearing my input maybe I dealt with the same challenge with 6 families now, or something like that could be beneficial to program delivery stuff.”

Hope valued and believed in the bringing together of front-line workers and managers and supervisors to increase communication among people in the hierarchical structure she believed existed within the organization.

PHN Ruby provided the following example of what she believed should transpire between herself and her work colleagues.

“But that’s because of my personality, that I really believe relationships are the foundation for everything we do. So if I don’t have a good relationship with my community partners, I’m not going to be able to work with them. If I don’t have a good relationship with my community home visitor, how am I ever going to promote her?”

Both PHNs and CHVs spoke about the importance of supportive relationships between each other as well as their managers. They believed this fostered a supportive working environment, guidance to implement effective care for mothers, respect for each other, and recognition for excellent work. These are all components of a strengths-based approach to care.

### 3.4. Negotiating Transitions of Families from PHNs to CHVs

The importance of communication in the process of transitioning families from a PHN who initially assessed a family, to a CHV who would continue to visit a family in their home was valued by both PHNs and CHVs. PHNs were the first point of contact for mothers and provided initial screening and then assessment of mothers to determine if they were eligible for the EHV Program. This process could involve a number of home visits. Once the program was offered to a mother, the PHN introduced the mother to a CHV who followed the mother or family in their home for up to three years. All of the PHNs and CHVs spoke about the relationship between the PHN and the CHV during the transfer of the mother from the PHN to the CHV. Opal, a PHN, described in the following quotation how she believed a client should be transitioned to a CHV and what she understood was part of the CHV role.

“So that transition to community home visitor support would be once everything is relatively stable in that client’s life so that they can learn more about parenting information, accessing information about growth and development for babies, be provided with support in terms of if employment is a goal that they have, or if finding affordable housing is a goal that they have, food security. So the community home visitor can help them kind of access healthy, nutritious foods, budgeting. So kind of mainly life skills that a family would need. I think that’s more of a CHVs’ role.”

In the next quotation, PHN Opal spoke about the importance of continuing with the communication between CHVs and PHNs after the transition and her frustration about how this practice was not formally supported.

“The clients will transition over to the CHVs, and then there’s really no formal structure in terms of how communication happens back and forth between Public Health and CHVs. So usually it just doesn’t’ happen. Now once in a while, there are some CHV’s I work with that are quite good at contacting me when issues or challenges arise. And there are others that I never hear from once I transition a client to them.”

Opal clearly identified a moment of tension or conflict in the way clients were transitioned from PHN visits to CHV visits. She was frustrated that there were no formal structures to support an ongoing connection between PHN’s and CHV’s.

The majority of PHNs and CHVs also spoke about their frustration about this gap in practice. All of the PHNS and CHVs were left to negotiate the transition in their own ways. Some reached out to each other and others did not. Without the formal structure of support, the majority of PHNs and CHVs felt their practice of transition was not recognized as important or legitimate and constructed an institutional practice whereby the relationship between CHVs and PHNs was hidden and not respected. The ways in which CHVs and PHNs negotiated their practice between each other during the transition included a subversive yet keenly felt relation of power that caused frustration for many. However, the lack of structure to support the transition did not stop Opal or the majority of CHVs and PHNs from working together. The CHVs and PHNs valued and believed in the importance of connecting and communicating in order to help families effectively transition from a PHN to a CHV. They continued to practice in this manner without support or recognition by management that eventually became an invisible part of their EHV practices that they believed was necessary to support the mothers. PHN Pearl shared her understanding about the lack of structure in the next example:

“I don’t know if there’s much of a structure. I think we individually as practitioners have our structures. But there’s no kind of checks and balances in terms of somebody checking to see what my caseloads are…so I don’t think there’s a formal structure.”

Below, PHN Opal described how she renegotiated her practice to ensure she connected with the CHV before the transition happened.

“And I’ve learned to change my practice so that before I contact a client, I will try and connect with the community home visitor and just to say is this person even still in the program or have they moved or what’s their new contact information? I’m coming up on 9 months and I need to reconnect with this client.”

Opal demonstrated her agency by continuing to connect with CHVs even though it was not formally supported by a structure within the hierarchical public health care system. Other CHVs and PHNs shared examples of complex negotiations that occurred within relationships in their practices that included PHNs and CHVs and systems beyond the EHV program. The systems surrounding home visiting practices included agencies external to the EHV system such as the Child Protective Services.

Transitioning a mother from a PHN to a CHV had been facilitated by the coordinator of the EHV program. When the position was eliminated, PHNs and CHVs felt the effects of not having someone to support the transition through communication between PHNs and CHVs. PHNs and CHVs demonstrated how they renegotiated how they transitioned mothers from PHNs to CHVs through creating their own way of communicating since the public health care institution did not provide an alternative approach when the coordinator role was cut from the EHV program delivery. Once again, PHNs and CHVs used their agency to create a practice of communication that supported what was needed in the transition of mothers in the EHV program. This finding supports the need to consider the roles of decision-makers within EHV and how they support practices of PHNs and CHVs in the future. Making program decisions without consulting PHNs and CHVs created tensions that were difficult for PHNs and CHVs in their practices.

The following quote from a PHN shows how they valued communication between each other in their practices: “Communication… coming together, sharing experiences, sharing practices, helping us understand how the nursing role has evolved…that’s what keeps our team strong and cohesive initially, was coming together and talking…”.

## 4. Discussion

Using a strengths-based approach when working with vulnerable populations has become an important part of the practice of PHNs and CHVs. Many nurse academics have spent their life’s work researching and building strengths-based approaches for health care practitioners [20,39,40] and strengths-based approaches can be found in a variety of provincial and national documents [41,42,43].

The importance of using strengths-based communication was a unique finding in this study as the experiences of the PHNs and CHVs demonstrated how relations of power both helped and hindered this foundational aspect of their practice. Standards and guidelines focusing on the importance of communication can be found in a variety of documents [41,42,43]. For example, community and public health experts suggest that strategic communication practices should be used to build relationships with individuals, families, communities, and groups. An environmental scan was completed by CHNC to develop competencies for Public Health Leadership in Canada. A literature review, online survey, and focus groups, generated the data for the scan. Specific skills identified in the scan for a public health leader include communicating clearly and transparently and supporting capacity building [44]. Promoting involvement, building partnerships, modeling as a mentor were also identified behaviors of a Public Health Leader. According to these Canadian authorities, communication is an important part of the relationships between workers such as PHNs, CHVs, and managers.

PHNs and CHVs in this study expressed the importance of effective, non-hierarchical, and supportive communication between themselves as well as their managers and supervisors. All participants gave examples of positive and effective communication. What was important to note in these examples was a common focus on strength-based interactions. Shifting the dominant hierarchical relationships to more equitable relations created a space for CHVs and PHNs to be able to debrief, feel supported, share their knowledge, and make changes within the program. The importance of this particular type of relational communication was more keenly expressed when they experienced a loss of this support. All PHNs and CHVs struggled with this loss for a variety of reasons. They all valued the ability of this coordinator to support, guide, and debrief with them. They also felt abandoned, as they had not been included in the decision to remove this position. They had to find other avenues for supportive, strengths-based communication with peers which turned out to be difficult. Many examples were shared by PHNs and CHVs about the effects of the changes. In particular, the way the decisions were made without their input left all PHNs and CHVs feeling excluded from the decision-making process.

A unique finding of this study was how CHVs struggled more with the binary relations of power due to their nonprofessional status of authority compared to PHNs. Despite the changes in the program, the PHNs and CHVs made sure that mothers living within vulnerability were not negatively affected. Compared to CHVs who were required to report to their supervisors, PHNs were not required to report, as their scope of practice supported them to work independently. Even though there was this difference, all PHNs and CHVs said they still needed the support of a PHN coordinator. All of the PHNs and CHVs shared how they negotiated the change and its impact on their practices and how they used their personal agency to re-negotiate the relations of power within their practices. The stories of how the PHNs and CHVs’ practices emerged within the changing structures and shifting organizations demonstrated the complexities of relations of power in their practices. For example, despite not having a coordinator to act as a liaison between them, PHNs and CHVs figured out ways to share and obtain the information they needed from each other, albeit it was not easy. PHN’s and CHV’s reaction to the change from this hierarchical relation of power is one Foucault refers to as the ability or agency of people to create an action or response to power that can be positive.

It was clearly evident from the examples shared in this study that the PHNs and CHVs valued their communication between each other and their managers. CHVs shared examples in the study of how communicating through reflecting with the PHN coordinator helped create a relationship where the relations of power between the CHV and PHN shifted so that there was a feeling of support for the CHV, where they would obtain advice or celebrate the way they had supported mothers living within vulnerability.

### Relevance to Clinical Practice

Understanding the complex communication practices in EHV emerged as a foundation of PHNs and CHVs day to day work with vulnerable mothers and their families. Guided reflective practice with a program coordinator was identified as an important support for the home visiting practices of EHVs in particular. PHNs independently decided what reflection strategies they utilized. However, they also valued reflecting with either a colleague or another team member. Providing emancipatory nursing practices to identify taken-for-granted norms and values that are experienced in practices can offer support for nurses as they reflect on the practices. In this study, the reflecting with the program coordinator offered CHVs the opportunity to talk about what they experienced in their EHV practices, and they felt a loss of personal support when it was gone as a result of the program change. This finding has an implication for EHV program policy/decision-makers to support the inclusion of reflection as a valued part of the everyday clinical practices of enhanced home visitors.

## 5. Conclusions

In all communication between people, there is a type of meaning that results. Sometimes the meaning is not understood immediately. CHVs shared how they understood the meaning of their communication with the coordinator very quickly after the position was no longer present. Sometimes, meaning is not directly shared between those who are in a relationship but the meaning of the loss of this person was verbally shared by almost all of the CHVs in the study. In this study, valued relationships were developed between PHNs and CHVs through communication and strategies they developed in their practices. The personal agency of PHNs and CHVs was evident in this study, however, a limitation of the study was the need to also uncover and understand the shifting structural and organizational changes that impacted the PHNs and CHVs agency.

## Data Availability

The data are not publicly available due to confidentiality agreements signed by participants in this study.
